# Multidrug-resistant enterobacteria in newborn dairy calves in Germany

**DOI:** 10.1371/journal.pone.0248291

**Published:** 2021-03-12

**Authors:** Jil Waade, Uwe Seibt, Walther Honscha, Fanny Rachidi, Alexander Starke, Stephanie Speck, Uwe Truyen

**Affiliations:** 1 Faculty of Veterinary Medicine, Institute of Animal Hygiene and Veterinary Public Health, Leipzig University, Leipzig, Germany; 2 Faculty of Veterinary Medicine, Institute of Pharmacology, Pharmacy and Toxicology, Leipzig University, Leipzig, Germany; 3 Faculty of Veterinary Medicine, Clinic for Ruminants and Swine, Leipzig University, Leipzig, Germany; Nitte University, INDIA

## Abstract

We studied the prevalence of extended-spectrum beta-lactamase (ESBL)-producing *Enterobacteriaceae* in dairy calves as part of a routine health check protocol. In addition, data regarding antimicrobial use (AMU), farm hygiene, and farm management were collected in order to identify possible risks for ESBL occurrence. Ten farms participated in the study with a median of 781 milking cows (319–1701). All calves investigated were younger than two weeks with an average age of 6.8 (±3.9) days. The farms were visited and samples were collected twice at an interval of 7–11 months. Faecal samples diluted 1:10, were plated onto *Brilliance*^TM^ ESBL agar in duplicates. After 24 hours at 37°C, colonies were counted and total colony forming units (cfu)/ml calculated. Bacteria species were identified biochemically. ESBL-production was phenotypically confirmed using the MICRONAUT-S β-Lactamases system. Additionally, antimicrobial susceptibility was tested using VITEK^®^ 2 technology. Phylotyping of *E*. *coli* isolates and screening for *bla* genes was performed by PCR. ESBL-producing enterobacteria were detected on all farms and 96.5% of calves investigated shed ESBL-positive bacteria. Of all ESBL-producing isolates, the majority were *E*. *coli* (92.9%), followed by *Enterobacter cloacae* (5.1%) and *Klebsiella pneumoniae* subsp. *pneumoniae* (2.0%). The majority of *E*. *coli* isolates was clearly assigned to phylogroup C (25.0%), followed by phylogroups A (15.2%) and E (14.1%). CTX-M group 1 was most frequently detected (80.4%). *E*. *cloacae* contained *bla*_CTX-M_ and *bla*_TEM_ or *bla*_SHV_. *K*. *pneumoniae* harboured *bla*_SHV_ only. Besides resistance to penicillins and cephalosporins, the majority of isolates was also resistant to one or more antibiotic classes, with a high proportion being resistant against fluoroqinolones. 52.5% of isolates were further characterised as threefold multidrug resistant gram-negative bacteria (3MDR-GNB) according to the German Commission for Hospital Hygiene and Infection Prevention. None of the isolates were 4MDR-GNB, i.e. none revealed carbapenem-resistance. Penicillins were the most frequently administered antibiotics to calves on most farms and were the predominant substance class at herd level on all farms. Overall, the number of calves treated prior to sampling was rather low (11.7%). Analyses of data regarding the farm management identified weaknesses in biosecurity and cleaning and disinfection. Besides beta-lactam antibiotics being the most commonly used antibiotics no other risk factors could be identified. In summary, the prevalence of ESBL-carriers in dairy calves was exceptionally high and should be motivation to develop strategies for the reduction of multidrug-resistant bacteria in farm animals.

## Introduction

Antimicrobial resistance is of worldwide concern. The WHO even goes as far as warning about a post-antibiotic era in which infections that are common now, will become life-threatening again [1]. The occurrence of multidrug-resistant bacteria in the community and in hospitals as well as in animal husbandry has increased rapidly over the last decades [2]. Especially the rise of multidrug-resistant Gram negative bacteria (MDR-GNB), with enterobacteria such as *Klebsiella* (*K*.) *pneumoniae* and *Escherichia* (*E*.) *coli*, is of growing concern and has been subject to many studies worldwide [3–5]. In Germany, MDR-GNB are further differentiated as so-called 3MDR-GNB (resistant against three classes of antibiotics, i.e. acylureidopenicillins, 3^rd^ and 4^th^ generation cephalosporins and fluoroquinolones) or 4MDR-GNB (resistant against four classes of antibiotics, i.e. carbapenems additionally to the aforementioned) [6]. In both, extended-spectrum beta-lactamases (ESBL) play an important role. ESBL are able to hydrolyse penicillins and cephalosporins including 3^rd^ and 4^th^ generation cephalosporins [7]. The latter are categorized by the WHO as critically important antimicrobials [8] as well as by the European Medical Association (EMA) as Category B (‘Restrict’) [9]. Resistance in ESBL-producing enterobacteria is predominantly determined by the plasmid-mediated beta-lactamase genes *bla*_SHV_, *bla*_TEM_ and *bla*_CTX-M_. In the 1990s and early 2000s, beta-lactamases of the TEM and SHV families dominated, whereas balance shifted towards the newly discovered family of CTX-M enzymes. Today, they are the most prevalent enzyme family with CTX-M-15 and CTX-M-1 most commonly isolated in humans and food animals [5]. The occurrence of ESBL-producing bacteria has been described to be highest in poultry, followed by cattle and pigs [10]. Prevalence in cattle vary greatly depending on age, production type and lactation stage. Watson et al. [11] reported a high proportion of newborn calves being positive for ESBL-producing *E*. *coli* (ESBLE). Incidence then declined from 97% at day 21 to less than 10% at day 161. Beef cattle are less likely to carry ESBLE compared to dairy cattle [11–13]. Shedding of ESBLE in cows is most prominent post-partum and during lactation [11]. Risk factors for the prevalence of ESBL-producing bacteria are believed to be feeding of waste milk that contains residues of antimicrobial substances [14, 15] and the use of 3^rd^ and 4^th^ generation cephalosporins. Management factors, such as an open herd policy and/or poor hygiene management, such as infrequent cleaning of feeding buckets, have also been identified as risk factors [16]. Although the data basis is steadily expanding, there is still a lack of information on the prevalence of ESBL in German dairy cattle [13, 17, 18], especially for the unique farm structure in the eastern part of Germany, where large herds are kept on few farms [19].

As part of a cooperation project entitled “Development of guidelines for the prevention of factorial diseases in cattle husbandry” we investigated the prevalence of MDR enterobacteria in dairy calves on ten farms. Data regarding antimicrobial use (AMU), farm hygiene, and management factors were collected and a possible link between farm hygiene, AMU, and MDR enterobacteria was examined.

## Materials and methods

### Study population

Ten dairy farms in Saxony, all within a radius of 100 km around the city of Chemnitz, volunteered to participate in the study and confirmed by written consent. All farms have been visited regularly by the Clinic for Ruminants and Swine in the preceding years as part of an integrated veterinary herd health care service or other scientific projects. The farms kept a median of 781 (319–1701) milking cows and a median of 36 (18–86) calves that were younger than two weeks. Calves were generally housed in individual pens or hutches for the first two weeks, either indoors (five farms) or outdoors (five farms). Calves on three farms were moved to group pens after 4–7 days. Waste milk was fed to the calves on seven of the ten farms. On one farm the milk was fed to all calves, on the remaining six farms only to bull calves. There was no data available on the proportion of waste milk containing antibiotic residues.

### Data collection and animal sampling

The farms were visited twice with an interval of 7–11 months from April 2018 to November 2019. During these visits an interview with the herd manager and an on-site inspection of the farm was carried out. The hygienic status of the farms was recorded and rated using a standardized questionnaire developed for dairy cattle [20]. The questions could either be answered with ‘no’ or ‘yes’ (translating to 0 or 3 scoring points) or in the fashion of ‘not fulfilled’, ‘partially fulfilled’ or ‘fulfilled’ (translating to 0, 1 or 3 scoring points). Weighted average ratios (in the following simply referred to as ratios) were calculated to describe the overall hygienic status on the farms as well as different aspects of dairy husbandry, such as biosecurity, feed and water hygiene, birth management and housing of cows and calves. Using scores between 0.00 to 3.00, the hygienic status of the farms was classified as poor (ratio 0.00–1.49, indicated by the colour red), medium (1.50–2.39, yellow) or good (2.40–3.00, green). Data on animal health and antimicrobial treatment were collected through animal scoring and the herd management program. The obtained information on AMU was collected in a database especially designed for this study and was further evaluated regarding gross amounts of used substances on herd level as well as for the different age groups.

On each farm, at each sampling day, faecal samples of 10 randomly selected calves, aged 0–14 days, were collected at each sampling day manually from the rectum or, if observed and clearly assignable to the individual, picked up from the ground. This adds up to a total of 200 samples. The samples were collected during routine health check-ups and transported at 4°C to the laboratory for further investigation. The presence of bovine coronavirus, bovine rotavirus, *Cryptosporidium parvum*, and *E*. *coli* K99 (F5) were examined using Speed V-Diar 4^TM^ (Virbac BVT, Bad Oldesloe, Germany) according to the manufacturer’s instructions. These results will be reported elsewhere. The ID number, age and gender of each calf were documented. The study was approved by the Animal Welfare Officer of the Veterinary Faculty at Leipzig University.

### Microbiological methods

All feacal samples were processed on the same day as collection. One gram of the sample was suspended in 9 ml NaCl-Trytpone broth (NaCl-T; pH 7.0; 1.0 g tryptone, 8.5 g NaCl ad 1000 ml *Aqua dest*.) and homogenized using a Laboratory Blender Stomacher 400 (Seward Limited, West Sussex, UK) for 60 sec. Homogenates were serially diluted 1:10 in NaCl-T. 100 μl of each suspension was plated in duplicates onto *Brilliance*^TM^ ESBL agar (Oxoid Deutschland GmbH/Thermo Scientific, Wesel, Germany) and incubated under aerobic conditions for 24 hours at 37°C. A pre-enrichment step was not performed. The colonies were counted and total colony forming units (cfu)/ml calculated. All morphologically different colonies from the samples of each farm were isolated and sub-cultured onto Columbia sheep blood agar (7% sheep blood; Oxoid Deutschland GmbH/Thermo Scientific) and Gassner agar (Sifin Diagnostics GmbH, Berlin, Germany). The bacterial species was identified biochemically using the RapID™ ONE System (Thermo Scientific, Wesel, Germany). If test results were inconclusive, isolates were sent to an external laboratory (Diagnosticum, Neukirchen, Germany) for species identification using MALDI TOF. All faecal samples and isolates were stored at -80°C after processing.

Further characterisation as described in the following, was performed on the first 100 isolates that were obtained.

### Susceptibility testing

ESBL-production was phenotypically confirmed using the commercially available MICRONAUT-S β-Lactamases system (Merlin Diagnostika GmbH, Bornheim-Hersel, Germany) according to the manufacturer’s instructions. Additionally, the isolates were sent to the Saxon State Laboratory for further testing of antimicrobial susceptibility using VITEK^®^ 2 technology (bioMérieux SA, Marcy-l’Étoile, France). The following substances were tested: ampicillin, ampicillin-sulbactam, amoxicillin, amoxicillin-clavulanic acid, piperacillin-tazobactam, cefuroxime, cefotiam, cefpodoxime, cefotaxime, ceftriaxone, ceftazidime, imipenem, meropenem, gentamicin, ciprofloxacin, levofloxacin, trimethoprim-sulfamethoxazole, and nitrofurantoin. Results were interpreted using clinical breakpoints for *Enterobacterales* defined by the European Committee on Antimicrobial Susceptibility Testing [21]. Furthermore, all isolates were classified as 3MDR-GNB or 4MDR-GNB according to the German Commission for Hospital Hygiene and Infection Prevention [6, 22].

### Determination of *bla*-genes

Isolates that proved to be phenotypically positive for ESBL-production were screened for the presence of *bla*_CTX-M_ groups 1 and 9, *bla*_TEM_, and *bla*_SHV_ using PCRs as described by Gröbner et al. [23] and Strauß et al. [24]. The primer sequences are listed in S1 Table. Prior to PCR, one to three colonies of each isolate were suspended in 200 μl phosphate-buffered saline (pH 7.2) and heated at 99°C for 10 minutes. The lysates were gently centrifuged and diluted 1:10 before being used for PCR. *E*. *coli* isolates 314/11 (CTX-M-2, phylogroup B1), 277/11 (TEM-52, phylogroup D), *Klebsiella pneumoniae* isolate 175/13 (SHV-1, CTX-M-15) and *Enterobacter cloacae* isolate 666/18 (CTX-M-9) were used as positive controls and were kindly provided by Yvonne Pfeifer, Robert Koch-Institute, Wernigerode, Germany.

### Phylotyping of *E*. *coli* isolates

Phylogroups were assigned using the quadruplex-PCR as described by Clermont et al. [25] which is able to detect strains belonging to group A, B1, B2, C, D, E, F, and clade I. Phylogroup assignment was carried out by scoring the presence or absence of the genes *arpA*, *chuA*, *yjaA*, TspE4.C2, and *trpA*. The primers used are described in S1 Table. The *E*. *coli* strains described above were also used as a control in the phylo-typing PCR. In addition, the following *E*. *coli* strains were used: 305/11 (phylogroup A), RL 45 (phylogroup C) and RL 72 (phylogroup E). The latter two were kindly provided by Dr. Jens Hammerl, Federal Institute for Risk Assessment (BfR), Berlin.

## Results and discussion

The objectives of this project were to study the prevalence of ESBL-producing enterobacteria in newborn calves on dairy farms, and to identify possible risk factors contributing to a high occurrence of ESBL-carriers. ESBL-producing enterobacteria were detected on all ten farms examined in the study. Of 200 faecal samples, 193 contained ESBL-producing enterobacteria which corresponded to a 96.5% prevalence of animals. This is much higher than reported in other studies performed in Germany, Switzerland, and Israel, which found an overall prevalence of 8.4%-56.2% and up to 26.2% in diseased calves [12, 13, 18, 26–28]. Major differences may relate to farm structures and farm practices, age of animals, and sampling on farms or at the slaughterhouse [12, 13, 28]. However, a similar amount (97.8%) of calves shedding ESBL-producers was found in the UK [13]. The highest prevalence of ESBL-E is usually found in young animals, notably suckling calves, which corresponds to our findings, but with increasing age ESBL-E prevalence gradually decreases [12, 13, 28, 29]. The average age of sampled calves in this study was 6.8 (±3.9) days. In our study animals older than 14 days were not sampled.

The average amount of ESBL-producing bacteria per g faeces was 1.7x10^9^ (±6.7x10^9^) cfu/g faeces which is more than reported elsewhere [30]. It is noteworthy that a valid comparison of different studies is difficult due to different study designs, sampling techniques, and screening protocols. Although we decided against a pre-enrichment step, the prevalence determined in our study was much higher compared to others that used a pre-enrichment step [13, 26, 27].

A total of 100 ESBL-producing isolates was further characterized. These included all 87 isolates from the first round of sampling as well as 13 isolates that were found on the first 3 farms during the second round of sampling. The majority were *E*. *coli* (92/100; 92.0%), followed by *Enterobacter* (*E*.) *cloacae* (5/100; 5.0%) and *Klebsiella* (*K*.) *pneumoniae* subsp. *pneumoniae* (2/100; 2.0%). One isolate was an AmpC-producing *Morganella morganae* and thus excluded from the study. This distribution is in accordance to two studies from Switzerland which reported *E*. *coli* being the predominant ESBL-producing species in faeces from cattle, whereas ESBL-producing *E*. *cloacae* were found to a much lesser extent [12, 26]. The occurrence of ESBL-producing *K*. *pneumoniae* in cattle faeces has only recently been described [31]. Of all faecal samples investigated, three contained more than one of the above mentioned ESBL-producing species. The majority of *E*. *coli* isolates was clearly assigned to phylogroup C (23/92; 25.0%), followed by phylogroups A (14/92; 15.2%) and E (13/92; 14.1%). Phylogroups B1, D, and F were found less frequently with ten (10.9%), eight (8.7%) and six (6.5%) isolates, respectively. None of the isolates belonged to phylogroup B2. For 18 out of 92 (19.6%) *E*. *coli* the unambiguous assignment to either phylogroup A or C was not possible using the PCRs of Clermont et al. [25]. These isolates are therefore referred to as members of phylogroup A/C. Overall, isolates belonging to phylogroups C, A/C, and A were predominant. This distribution is in contrast to other studies where phylogroups A and B1 were dominantly found in ESBLE isolates from healthy animals [18, 32–36]. The main reason for the differences compared to other studies might be that the PCR-protocols used elsewhere may not discriminate between phylogroups A and C [18, 33, 35, 36]. However, differences in phylogroup distribution might also be attributed to geographic location, climate, feeding habits or other factors [37].

We screened 99 ESBL-producing isolates for the presence of *bla* genes. Detailed results can be obtained from Table 1. The majority of *E*. *coli* isolates (75/92, 81.5%) harboured *bla*_CTX-M_ genes, with CTX-M group 1 predominating (80.4%). This is consistent with other German and European studies in which most frequently CTX-M group 1 followed by CTX-M group 9 were detected [13, 18, 27, 35, 38]. CTX-M group 9 was detected in five *E*. *coli* either as the only *bla* gene (n = 1) or in combination with other *bla* genes (n = 4). Only one isolate did not belong to either group 1 or 9. In 45 *E*. *coli* (48.9%), *bla*_CTX-M_ was found in combination with *bla*_TEM_. Overall, the prevalence of *bla*_TEM_ (61.9%) in *E*. *coli* either in combination with *bla*_CTX-M_ or alone was higher compared to other studies [33, 39]. Four *E*. *coli* (4.3%) were negative for CTX-M, TEM, and SHV. Of the ESBL-producing *E*. *cloacae* and *K*. *pneumoniae*, one *E*. *cloacae* contained *bla*_CTX-M_ and *bla*_TEM_ whereas the remaining four and the two *K*. *pneumoniae* harboured *bla*_SHV_. Studies on EBSL-producing enterobacteria other than *E*. *coli* in animals are scarce. In contrast to our findings, *bla*_SHV_ was the dominant gene in *E*. *coli* compared to *K*. *pneumoniae* in a recent South African study [31]. To the authors best knowledge there is only one report on *E*. *cloacae* carrying *bla*_SHV_ and *bla*_TEM_ isolated from a sheep lamb in Switzerland [27].

**Table 1:** Distribution of *bla* genes.

		Number of isolates (%)		
	total (n = 99)	*E*. *coli* (n = 92)	*E*. *cloacae* (n = 5)	*K*. *pneumoniae* (n = 2)
CTX-M-1 group + TEM	43 (43.4)	42 (45.7)	1 (20.0)	0 (0.0)
CTX-M-1 group	28 (28.3)	28 (30.4)	0 (0.0)	0 (0.0)
TEM	12 (12.1)	12 (13.0)	0 (0.0)	0 (0.0)
CTX-M-1 group + CTX-M-9 group + TEM	3 (3.0)	3 (3.3)	0 (0.0)	0 (0.0)
CTX-M-9 group	1 (1.0)	1 (1.1)	0 (0.0)	0 (0.0)
CTX-M-1 group + CTX-M-9 group	1 (1.0)	1 (1.1)	0 (0.0)	0 (0.0)
Other CTX-M group	1 (1.0)	1 (1.1)	0 (0.0)	0 (0.0)
SHV	6 (6.1)	0 (0.0)	4 (80.0)	2 (100.0)
Not determinable	4 (4.0)	4 (4.3)	0 (0.0)	0 (0.0)

We further characterised ESBL-producing bacteria for multidrug-resistance (i.e. as 3MDR-GNB or 4MDR-GNB). The definition given by the German Commission for Hospital Hygiene and Infection Prevention [6] is part of a national guideline which is used to guide infection prevention and control measures in human clinical settings [40]. Acknowledging the One Health concept and the risk of bacterial transmission between animals and humans in either direction, data on the occurrence of these MDR bacteria are of high importance for public health and animal health risk assessment. Besides resistance to penicillins and cephalosporins, 60 ESBL-producing isolates (60.6%) were additionally resistant to one or more antibiotic classes as determined by VITEK^®^ 2 (Table 2, S2 Table) which is in accordance to other studies in cattle [13, 27, 35, 41] and other food animals [27, 35, 42]. Concordant to Schmid et al. [13] and Eisenberger et al. [43], a high proportion of isolates (46.5%) was also resistant to fluoroquinolones. Most probably quinolone resistance is plasmid-mediated by genes co-located to *bla*_CTX-M_ genes. However, even without plasmid-encoded insensitivity a strong association between quinolone resistance and ESBL-production has been described [44, 45]. Fiftytwo isolates (52.5%) were further classified as 3MDR-GNB. This finding was similar to results from ESBL-producing *E*. *coli* and *K*. *pneumoniae* isolated from humans [46]. None of the isolates proved to be 4MDR-GNB, i.e. all were susceptible to imipenem and meropenem (Table 2) which is contrary to a study from the UK that reported an unstable imipenem-resistant phenotype in 1.2% of their *E*. *coli* isolates [41]. In Europe, screening for carbapenem-resistance in livestock is still underrepresented and prevalence in European countries was reported as <1% [47]. In Germany, carbapenem-resistant *Enterobacteriaceae* were mainly associated with swine and chicken whereas only a single report on ertapenem-resistance in *E*. *coli* from a beef cattle farm is published [13, 47].

**Table 2:** Species distribution and antimicrobial resistance profile of the 99 ESBL-producing isolates.

Resistance against		Number of isolates (%)		
	total n (%)	*E*. *coli* (n = 92)	*E*. *cloacae* (n = 5)	*K*. *pneumoniae* (n = 2)
Ampicillin, amoxicillin	99 (100.0)	92 (100.0)	5 (100.0)	2 (100.0)
Ampicillin-sulbactam, amoxicillin-clavulanic acid	88 (88.9)	85 (92.4)	1 (20.0)	2 (100.0)
Piperacillin-tazobactam	4 (4.0)	4 (4.3)	0 (0.0)	0 (0.0)
Cefuroxime, cefotiam	99 (100.0)	92 (100.0)	5 (100.0)	2 (100.0)
Cefpodoxime proxetil	99 (100.0)	92 (100.0)	5 (100.0)	2 (100.0)
Cefotaxime, ceftriaxone	99 (100.0)	92 (100.0)	5 (100.0)	2 (100.0)
Ceftazidime	16 (16.2)	12 (13.0)	4 (80.0)	0 (0.0)
Imipenem	0 (0.0)	0 (0.0)	0 (0.0)	0 (0.0)
Meropenem	0 (0.0)	0 (0.0)	0 (0.0)	0 (0.0)
Gentamicin	25 (25.3)	22 (23.9)	1 (20.0)	2 (100.0)
Ciprofloxacin	46 (46.5)	43 (46.7)	1 (20.0)	2 (100.0)
Levofloxacin	43 (43.4)	43 (46.7)	0 (0.0)	0 (0.0)
Trimethoprim-sulfamethoxazole	45 (45.5)	42 (45.7)	1 (20.0)	2 (100.0)
Nitrofurantoin	1 (1.1)	1 (1.1)	N/A	N/A

N/A–not applicable.

The gross amounts (in g) of antimicrobial substances administered on farm level are shown in S3 Table. In contrast to other European countries such as Denmark or The Netherlands, reporting of AMU in dairy calves is not mandatory in Germany according to current jurisdiction [48]. Therefore, the results presented here highly depended on the quality of documentation at farm level. Unfortunately, data regarding the use of antibiotics on one farm could not be evaluated due to inadequate documentation. Of 180 calves on the other farms, for which information on treatment was available, 21 (11.7%) received antibiotic treatment prior to sampling, four of which were treated twice. On two farms none of the sampled calves were treated before. Substances administered to calves and their corresponding indications are given in Table 3. There was no correlation between the treatment of a calf with antibiotics and their status as ESBL-carrier (P = 0.7765).

**Table 3:** Antibiotic treatment of calves investigated and corresponding therapeutic indications.

Antibiotic substance	Indication				Total treatments
	Pneumonia	Enteritis	Omphalitis	Fever	
Amoxicillin	4	1	5	0	**10**
Marbofloxacin	3	1	0	1	**5**
Benzylpenicillin	0	0	3	0	**3**
Colistin	0	1	1	0	**2**
Tulathromycin	2	0	0	0	**2**
Sulfadoxine/trimethoprim	0	0	2	0	**2**
Florfenicol	1	0	0	0	**1**
**Total treatments**	**10**	**3**	**11**	**1**	**25**

A total of 21 animals was treated. Four calves were treated twice. The numbers given in the table indicate the number of treatments independent of the duration of therapy.

**Fig 1:**
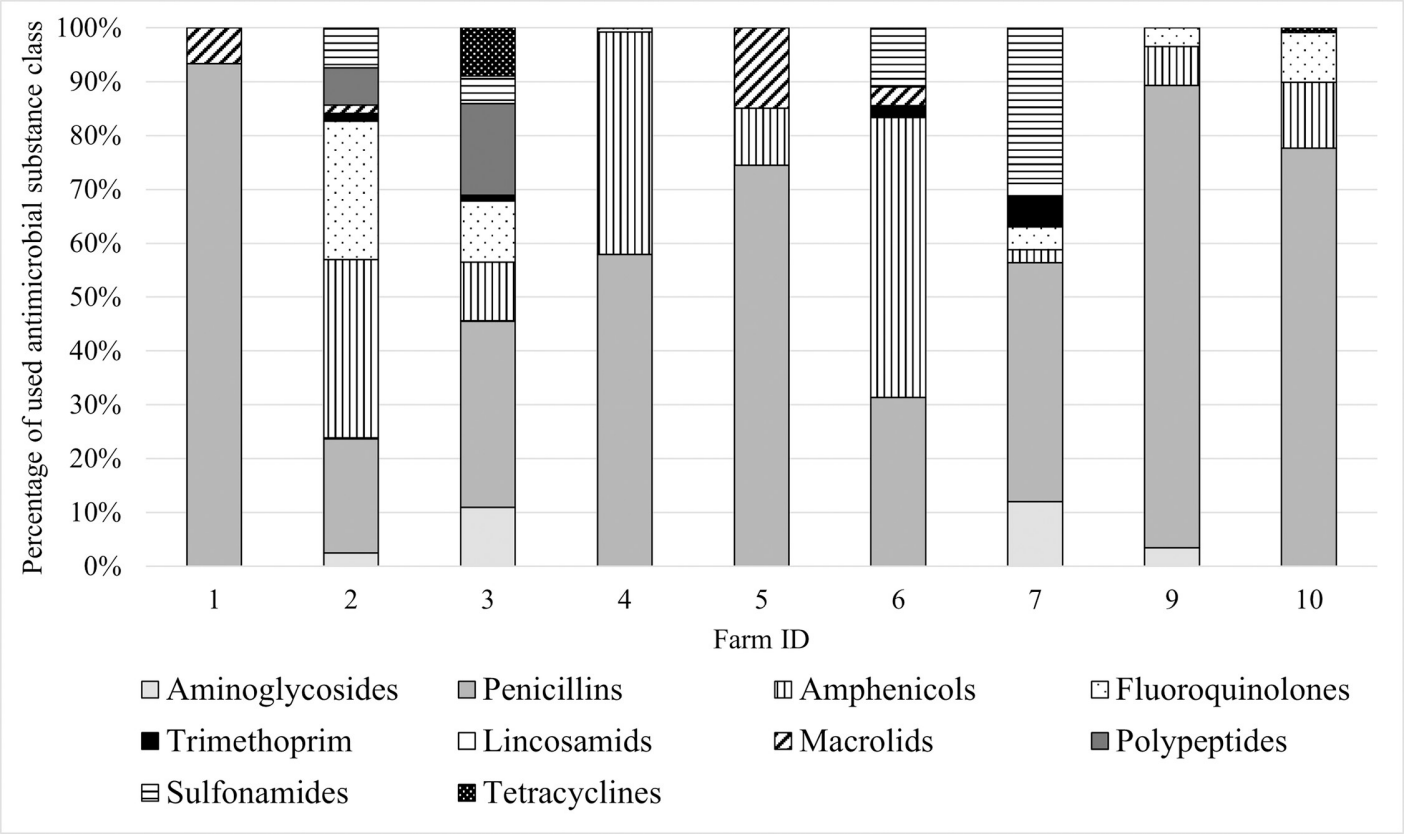
Proportional use of antibiotic substance classes on the 9 farms in newborn calves. Gross amounts in g were used as data basis.

Of all thirteen antibiotic substance classes documented, 8–11 were used on herd level on all farms, whereas for calves the use was less diverse (2–9 substance classes). Penicillins were most frequently used in calves on all but two farms (Farm 2 and 6), which mainly administered amphenicols (Fig 1). On herd level, penicillins were the predominant substance class on all farms. Similar results were reported for dairy herds in the UK [49] and cattle in Germany [50]. However, van Rennings et al. [50] did not differentiate between production type or age group. Fluoroquinolones were used on six farms which might account for the high amount of resistant isolates in our study. Cephalosporins were not administered to calves on any of the farms. A comparison of AMU among the participating farms regarding gross amounts of antibiotic substance was not possible because a reliable treatment index could not be calculated from the animal numbers obtainable through the herd management program.

The feeding of waste milk to the sampled calves had no statistically significant effect on their status as ESBL-carrier (P = 0.1787).

The scores obtained from hygiene analyses during both visits at each farm are shown in Table 4. Scores for overall hygiene ranged between 1.90 and 2.64. On average, the highest scores were achieved for the subsections ‘barn climate’ and ‘milking’ whereas the subsections ‘biosecurity’ and ‘cleaning and disinfection’ were assessed poorest. Because of the nearly 100% prevalence of ESBLE no correlation to any possible risk factor could be evaluated.

**Table 4:** Results of the hygiene analyses for the 10 farms during both farm visits.

	Farm ID (no. of visits)																			
	1		2		3		4		5		6		7		8		9		10	
	(1)	(2)	(1)	(2)	(1)	(2)	(1)	(2)	(1)	(2)	(1)	(2)	(1)	(2)	(1)	(2)	(1)	(2)	(1)	(2)
**Overall scoring**	**2.28**	**2.31**	**2.15**	**2.16**	**2.30**	**2.25**	**2.28**	**2.31**	**2.15**	**2.14**	**1.91**	**1.90**	**2.64**	**2.42**	**2.25**	**2.24**	**2.38**	**2.38**	**2.24**	**2.26**
Biosecurity	1.62	1.62	1.84	1.84	1.79	1.83	1.94	1.94	1.52	1.52	0.84	0.84	2.20	2.20	2.00	2.00	1.66	1.66	1.60	1.60
Cleaning and disinfection	1.84	1.84	2.05	2.05	2.01	2.01	2.04	2.08	1.96	1.96	1.64	1.62	2.27	2.27	2.19	2.19	1.74	1.74	1.95	1.95
Feeding and water	2.10	1.96	1.69	1.85	1.84	1.84	1.82	1.98	2.01	1.97	1.59	1.59	2.19	2.32	2.21	2.21	2.51	2.42	2.08	2.08
Carcasses, waste products, disinfestation	2.16	2.16	1.58	1.67	2.32	2.32	2.34	2.42	2.01	1.79	1.72	1.72	2.21	2.32	1.98	1.98	2.49	2.49	2.14	2.14
Animal housing	2.38	2.34	2.21	2.24	1.96	1.93	1.77	1.83	2.21	2.25	1.66	1.66	2.38	2.32	1.99	1.94	2.39	2.39	2.06	2.06
Barn climate	2.73	2.83	2.51	2.47	2.47	2.35	2.73	2.77	2.74	2.79	2.63	2.43	2.96	2.73	2.96	2.96	2.64	2.64	2.57	2.87
Animal transport	2.56	2.56	2.06	2.06	2.88	2.88	2.69	2.69	2.63	2.63	1.81	1.81	2.13	2.13	2.81	2.81	2.81	2.81	2.31	2.31
Quarantine and sick pens	2.13	2.13	2.07	2.07	2.67	2.67	2.47	2.47	2.13	2.13	2.07	2.07	2.67	2.67	2.07	2.07	2.47	2.47	1.93	1.93
Insemination and birthing	2.18	2.48	2.23	2.23	2.30	2.30	2.35	2.32	2.04	2.04	2.12	2.12	2.47	2.29	1.71	1.71	2.30	2.30	2.57	2.57
Milking	2.55	2.55	2.43	2.43	2.45	2.45	2.54	2.54	2.50	2.50	2.20	2.20	2.79	2.79	2.48	2.48	2.73	2.73	2.42	2.42
Farm management	2.60	2.60	2.35	2.35	2.75	2.75	2.55	2.55	1.98	1.98	2.35	2.35	2.55	2.55	2.60	2.60	2.60	2.60	2.60	2.60

Ratios <1.50 indicate poor hygiene (highlighted in red); ratios 1.50–2.39 indicate medium hygiene (highlighted in yellow); ratios ≥2.40 indicate good hygiene (highlighted in green).

In other studies, several factors contributing to a higher occurrence of ESBLE have been identified: e.g. cleanliness of calf housings and calving pens, crowdedness, not applying teat sealants in cows at dry off, parental treatment of clinical mastitis, feeding milk replacer to calves, or the use of third- and fourth-generation cephalosporins [15, 28, 35, 51, 52]. The latter, however, is discussed controversially [52]. Moreover, pig farms located within a small radius of the cattle barn have been associated with higher odds of ESBL occurrence which might be attributed to airborne dust-bound ESBLE [52, 53].

One of the aims of this study was to elucidate possible links between farm hygiene, AMU, and MDR enterobacteria. In our study, we determined a 100% ESBL prevalence at farm level. On all farms beta-lactams were the most commonly used antibiotics, but because of the 100% prevalence of ESBLE at farm level no correlation to any possible risk factor could be evaluated.

The small sample number and the restriction to calves younger than two weeks can be seen as limitations of the study. Due to the fact that only the first 100 isolates obtained across the project were further analysed, which represented all ten farms only in the first sampling, whereas in the second sampling only farms 1–3 were included, an overrepresentation of these farms cannot be excluded. The occurrence of ESBL-producing enterobacteria is most likely a multifactorial process. Hence, a longitudinal study including AMU and other relevant management factors, health and ESBLE status of cows and calves is required to identify risk factors contributing to a high prevalence of ESBLE in dairy herds. The high percentage of ESBLE-carriage in calves reported here, highlights the overall need to develop strategies for the reduction of multidrug-resistant enterobacteria in newborn calves.

## Conclusions

Our study revealed a 100% prevalence of ESBL-producing enterobacteria in calves at farm level and a prevalence of 96.5% at the individual level. Beta-lactam antibiotics were the most frequently used antimicrobials on the participating farms. This may have contributed to the persistence of ESBL at farm level and possibly to a co-selection of resistance against other antimicrobial substances. Although ESBLE-prevalence has been described to decrease with increasing age of cattle, the high prevalence as well as the high number of ESBL-producing bacteria that are shed, require strategies to prevent the entry of ESBLE into the calf rearing system at an early stage such as prudent use of antimicrobials during drying off and diligent hygiene in calving pens and calf housing. Further investigation is needed, to define the entry point(s) of ESBLE into calf rearing.

## Supporting information

S1 Table Primer sequences and sizes of PCR products used for the determination of *bla*-genes and the extended quadruplex phylo-typing method. (DOCX)

S2 Table Minimum inhibitory concentrations (mg/L) determined by VITEK^®^ 2 technology for 99 ESBL-producing isolates. (DOCX)

S3 Table Gross amounts of antibiotic substance classes (in g) used on the farms in total and in newborn calves. (DOCX)

## References

[1] WHO. 2014. Antimicrobial Resistance: Global Report on Surveillance. World Health Organization, Geneva, 232 pp

[2] Levy SB. Microbial resistance to antibiotics: an evolving and persistent problem. Lancet. 1982; 320 (8289), 83–88. 10.1016/S0140-6736(82)91701-9 6123819

[3] Carattoli A. Animal reservoirs for extended spectrum beta-lactamase producers. Clin. Microb. Infect. 2008; 14, 117–123. 10.1111/j.1469-0691.2007.01851.x 18154535

[4] Coque TM, Baquero F, Canton R. Increasing prevalence of ESBL-producing *Enterobacteriaceae* in Europe. Euro Surveill. 2008; 13 (47), 19044. 10.2807/ese.13.47.19044-en 19021958

[5] Liebana E, Carattoli A, Coque TM, Hasman H, Magiorakos AP, Mevius D et al. Public health risks of enterobacterial isolates producing extended-spectrum β-lactamases or AmpC β-lactamases in food and food-producing animals: an EU perspective of epidemiology, analytical methods, risk factors, and control options. Clin. Infect. Dis. 2013; 56 (7), 1030–1037. 10.1093/cid/cis1043 23243183

[6] Commission for Hospital Hygiene and Infection Prevention (KRINKO) at the Robert Koch Institute (RKI). [Hygiene measures for infection or colonization with multidrug-resistant gram-negative bacilli. Commission recommendation for hospital hygiene and infection prevention (KRINKO) at the Robert Koch Institute (RKI)]. Bundesgesundheitsblatt Gesundheitsforschung Gesundheitsschutz. 2012; 55:1311–54, Erratum in: Bundesgesundheitsblatt Gesundheitsforschung Gesundheitsschutz 2013;56:1342 10.1007/s00103-012-1549-5 23011096

[7] Bonnet R. Growing group of extended-spectrum ß-lactamases: the CTX-M enzymes. Antimicrob. Agents and Chemotherapy. 2004; 48 (1), 1–14. 10.1128/AAC.48.1.1 PMC310187 14693512

[8] WHO. Critically important antimicrobials for human medicine– 5th rev., World Health Organisation, Geneva. 2017; 48 pp

[9] EMA/CVMP/CHM. Categorisation of antibiotics in the European Union. 2019 [cited 30 August 2020]. Available from: https://www.ema.europa.eu/en/documents/report/categorisation-antibiotics-european-union-answer-request-european-commission-updating-scientific_en.pdf

[10] Hille K, Fischer J, Falgenhauer L, Sharp H, Brenner GM, Kadlec K et al. [On the occurence of Extended-spectrum- and AmpC-beta- lactamase-producing *Escherichia coli* in livestock: results of selected European studies]. Berl. Münch. Tierärztl. Wochenschr. 2014; 127 (9/10), 403–411. 10.2376/0005-9366-127-403 25868168

[11] Watson E, Jeckel S, Snow L, Stubbs R, Teale C, Wearing H et al. Epidemiology of extended spectrum beta-lactamase *E*. *coli* (CTX-M-15) on a commercial dairy farm. Vet. Microbiol. 2012; 154 (3–4), 339–346. 10.1016/j.vetmic.2011.07.020 21840142

[12] Reist M, Geser N, Hächler H, Schärrer S, Stephan R. ESBL-producing *Enterobacteriaceae*: occurrence, risk factors for fecal carriage and strain traits in the Swiss slaughter cattle population younger than 2 years sampled at abattoir level. PloS one. 2013; 8 (8), e71725. 10.1371/journal.pone.0071725 23977126 PMC3748101

[13] Schmid A, Hörmansdorfer S, Messelhäusser U, Käsbohrer A, Sauter-Louis C, Mansfeld R. Prevalence of extended-spectrum β-lactamase-producing *Escherichia coli* on Bavarian dairy and beef cattle farms. Appl. Environ. Microbiol. 2013; 79 (9), 3027–3032. 10.1128/AEM.00204-13 23455336 PMC3623142

[14] Aust V, Knappstein K, Kunz HJ, Kaspar H, Wallmann J, Kaske M. Feeding untreated and pasteurized waste milk and bulk milk to calves: effects on calf performance, health status and antibiotic resistance of faecal bacteria. J. Anim. Physiol. Anim. Nutr. 2013; 97 (6), 1091–1103. 10.1111/jpn.12019 23205592

[15] Gonggrijp MA, Santman-Berends IMGA, Heuvelink AE, Buter GJ, van Schaik G, Hage JJ et al. Prevalence and risk factors for extended-spectrum β-lactamase- and AmpC-producing *Escherichia coli* in dairy farms. J. Dairy Sci. 2016; 99 (11), 9001–9013. 10.3168/jds.2016-11134 27638264

[16] Snow LC, Warner RG, Cheney T, Wearing H, Stokes M, Harris K et al. Risk factors associated with extended spectrum beta-lactamase *Escherichia coli* (CTX-M) on dairy farms in North West England and North Wales. Prev. Vet. Med. 2012; 106 (3–4), 225–234. 10.1016/j.prevetmed.2012.03.009 22552330

[17] Büchter B. [Occurence and characterisation of extended-spectrum ß-lactamase (ESBL)-producing *Escherichia coli* in food-producing animals]. Dissertation. Freie Univ. Berlin. 2010. Available from: https://refubium.fu-berlin.de/bitstream/handle/fub188/956/online.pdf?sequence=1&isAllowed=y&save=y

[18] Michael GB, Kaspar H, Siqueira AK, Freitas Costa E de, Corbellini LG, Kadlec K et al. Extended-spectrum β-lactamase (ESBL)-producing *Escherichia coli* isolates collected from diseased food-producing animals in the GERM-Vet monitoring program 2008–2014. Vet. Microbiol. 2017; 200, 142–150. 10.1016/j.vetmic.2016.08.023 27634182

[19] Merle R, Busse M, Rechter G, Meer U. [Regionalisation of Germany by data of agricultural structures]. Berl. Münch. Tierärztl. Wochenschr. 2012; 8 (1–2), 52–59. 10.2376/0005-9366-125-52 22372325

[20] Müller KE, Englisch A, Tautenhahn A, Gäbler E, Forkmann A, Rösler U et al. [Development and testing of an evaluation system for hygiene, animal welfare and animal health on cattle farms]. Schriftenreihe des LfULG, Heft 5/2016. ISBN: 1867–2868

[21] EUCAST. New S, I and R definitions. ESCMID—European Society of Clinical Microbiology and Infectious Diseases. 2019 [cited 19 April 2020]. Available from: http://www.eucast.org/newsiandr/

[22] Commission for Hospital Hygiene and Infection Prevention (KRINKO) at the Robert Koch Institute (RKI). [Supplement to the commission recommendation for hospital hygiene and infection prevention (KRINKO) at the Robert Koch Institute (RKI) ‚Hygiene measures for infection or colonization with multidrug-resistant gram-negative bacilli‘ (2012) in the context of the redefined susceptibility testing category I by EUCAST: consequences for the definition of MDR-GN]. Epid. Bull. 2019;9:82 – 83. 10.25646/5916

[23] Gröbner S, Linke D, Schütz W, Fladerer C, Madlung J, Autenrieth IB et al. Emergence of carbapenem-non-susceptible extended-spectrum beta-lactamase-producing *Klebsiella pneumoniae* isolates at the university hospital of Tübingen, Germany. J. Med. Microb. 2009; 58 (7), 912–922. 10.1099/jmm.0.005850-0 19502377

[24] Strauß LM, Dahms C, Becker K, Kramer A, Kaase M, Mellmann A. Development and evaluation of a novel universal β-lactamase gene subtyping assay for blaSHV, blaTEM and blaCTX-M using clinical and livestock-associated *Escherichia coli*. J. Antimicrob. Chemother. 2015; 70 (3), 710–715. 10.1093/jac/dku450 25414200

[25] Clermont O, Christenson JK, Denamur E, Gordon DM. The Clermont *Escherichia coli* phylo-typing method revisited: improvement of specificity and detection of new phylo-groups. Environ. Microbiol. Rep. 2013; 5 (1), 58–65. 10.1111/1758-2229.12019 23757131

[26] Geser N, Stephan R, Kuhnert P, Zbinden R, Kaeppeli U, Cernela N et al. Fecal carriage of extended-spectrum β-lactamase-producing *Enterobacteriaceae* in swine and cattle at slaughter in Switzerland. J. Food Prot. 2011; 74 (3), 446–449. 10.4315/0362-028X.JFP-10-372 21375882

[27] Geser N, Stephan R, Hächler H. Occurrence and characteristics of extended-spectrum β-lactamase (ESBL) producing *Enterobacteriaceae* in food producing animals, minced meat and raw milk. BMC Vet. Res. 2012; 8, 21. 10.1186/1746-6148-8-21 22397509 PMC3319423

[28] Adler A, Sturlesi N, Fallach N, Zilberman-Barzilai D, Hussein O, Blum SE et al. Prevalence, risk factors, and transmission dynamics of extended-spectrum-β-lactamase-producing *Enterobacteriaceae*: a national survey of cattle farms in Israel in 2013. J. Clin. Microbiol. 2015; 53(11), 3515–3521. 10.1128/JCM.01915-15 26311861 PMC4609711

[29] Brunton LA, Reeves HE, Snow LC, Jones JR. A longitudinal field trial assesing the impact of feeding wastemilk containing antibiotic residues on the prevalence of ESBL-producing *Escherichia coli* in calves. Prev. Vet. Med. 2014; 117 (2), 403–412. 10.1016/j.prevetmed.2014.08.005 25172121

[30] Tetens JL, Billerbeck S, Schwenker JA, Hölzel CS. Short communication: Selection of extended-spectrum β-lactamase-producing *Escherichia coli* in dairy calves associated with antibiotic dry cow therapy-A cohort study. J. Dairy Sci. 2019; 102 (12), 11449–11452. 10.3168/jds.2019-16659 31629516

[31] Montso KP, Dlamini SB, Kumar A, Ateba CN, Garcia-Perdomo HA. Antimicrobial Resistance Factors of Extended-Spectrum Beta-Lactamases Producing *Escherichia coli* and *Klebsiella pneumoniae* Isolated from Cattle Farms and Raw Beef in North-West Province, South Africa. BioMed. Res. Int. 2019; Article ID 4318306. 10.1155/2019/4318306 31915693 PMC6935440

[32] Valat C, Auvray F, Forest K, Métayer V, Gay E, Peytavin de Garam C et al. Phylogenetic grouping and virulence potential of extended-spectrum-β-lactamase-producing *Escherichia coli* strains in cattle. Appl. Environ. Microb. 2012; 78 (13), 4677–4682. 10.1128/AEM.00351-12 22522692 PMC3370483

[33] Valentin L, Sharp H, Hille K, Seibt U, Fischer J, Pfeifer Y et al. Subgrouping of ESBL-producing *Escherichia coli* from animal and human sources: an approach to quantify the distribution of ESBL types between different reservoirs. Int. J. Med. Microbiol. 2014; 304 (7), 805–816. 10.1016/j.ijmm.2014.07.015 25213631

[34] Coura FM, Araújo Diniz S de, Mussi JMS, Silva MX, Lage AP, Heinemann MB. Characterization of virulence factors and phylogenetic group determination of *Escherichia coli* isolated from diarrheic and non-diarrheic calves from Brazil. Folia Microbiol. (Praha). 2017; 62 (2), 139–144. 10.1007/s12223-016-0480-9 27787756

[35] Hille K, Felski M, Ruddat I, Woydt J, Schmid A, Friese A. Association of farm-related factors with characteristics profiles of extended-spectrum β-lactamase-/plasmid-mediated AmpC β-lactamase-producing *Escherichia coli* isolates from German livestock farms. Vet. Microbiol. 2018; 223, 93–99. 10.1016/j.vetmic.2018.07.022 30173759

[36] Umair M, Mohsin M, Ali Q, Qamar MU, Raza S, Ali A et al. Prevalence and genetic relatedness of extended spectrum-β-lactamase-producing *Escherichia coli* among humans, cattle, and poultry in Pakistan. Microb. Drug Resist. 2019; 25 (9), 1374–1381. 10.1089/mdr.2018.0450 31268408

[37] De Castro Stoppe N, Silva JS, Carlos C, Sato MIZ, Saraiva AM, Ottoboni LMM et al. Worldwide phylogenetic group patterns of *Escherichia* coli from commensal human and wastewater treatment plant isolates. Front. Microbiol. 2017; 8, 10.3389/fmicb.2017.02512 29312213 PMC5742620

[38] Ewers C, Bethe A, Semmler T, Guenther S, Wieler LH. Extended-spectrum β-lactamase-producing and AmpC-producing *Escherichia coli* from livestock and companion animals and their putative impact on public health: a global perspective. Clin. Microb. Infect. 2012; 18 (7), 646–655. 10.1111/j.1469-0691.2012.03850.x 22519858

[39] Käsbohrer A, Bakran-Lebl K, Irrgang A, Fischer J, Kämpf P, Schiffmann A et al. Diversity in prevalence and characteristics of ESBL/pAmpC producing *E*. *coli* in food in Germany. Vet. Microbiol. 2019; 233, 52–60. 10.1016/j.vetmic.2019.03.025 31176413

[40] Wolfensberger A, Kuster SP, Marchesi M, Zbinden R, Hombach M. The effect of varying multidrug-resistence (MDR) definitions on rates of MDR gram-negative rods. Antimicrob. Resist. Infect. Control. 2019; 8, 193. 10.1186/s13756-019-0614-3 31798839 PMC6883537

[41] Ibrahim DR, Dodd CER, Stekel DJ, Ramsden SJ, Hobman JL. Multidrug resistant, extended spectrum β-lactamase (ESBL)-producing *Escherichia coli* isolated from a dairy farm. FEMS Microbiol. Ecol. 2016; 92 (4). 10.1093/femsec/fiw013 26850161

[42] Faccone D, Moredo FA, Giacoboni GI, Albornoz E, Alarcón L, Nievas VF et al. Multidrug-resistant *Escherichia coli* harbouring mcr-1 and blaCTX-M genes isolated from swine in Argentina. J. Glob. Antimicrob. Resist. 2019; 18, 160–162. 10.1016/j.jgar.2019.03.011 30926466

[43] Eisenberger D, Carl A, Balsliemke J, Kämpf P, Nickel S, Schulze G et al. Molecular characterization of extended-spectrum β-lactamase-producing *Escherichia coli* isolates from milk samples of dairy cows with mastitis in Bavaria, Germany. Microb. Drug Resist. 2018; 24 (4), 505–510. 10.1089/mdr.2017.0182 28953418

[44] Paterson DL, Bonomo RA. Extended-spectrum β-lactamases: a clinical update. Clin. Microbiol. Rev. 2005; 18(4), 657–686. 10.1128/CMR.18.4.657-686.2005 16223952 PMC1265908

[45] Jacoby GA, Strahilevitz J, Hooper DC. Plasmid-mediated quinolone resistance. In: Tolmasky M, Alonso JC, editors. Plasmids. Biology and impact in biotechnology and discovery. ASM Press, Washington, DC. 2015. pp. 475–503

[46] Pfeifer Y, Eller C, Leistner R, Valenza G, Nickel S, Guerra B. [ESBL producer as human pathogens and the zoonotic reservoir]. Hyg. Med. 2013; 38 (7/8), 294–299

[47] Köck R, Daniels-Haardt I, Becker K, Mellmann A, Friedrich AW, Mevius D et al. Carbapenem-resistant *Enterobacteriaceae* in wildlife, food-producing, and companion animals: a systematic review. Clin. Microbiol. Infect. 2018; 24 (12), 1241–1250. 10.1016/j.cmi.2018.04.004 29654871

[48] Hommerich K, Ruddat I, Hartmann M, Werner N, Käsbohrer A, Kreienbrock L. Monitoring antibiotic usage in German dairy and beef cattle farms—A longitudinal analysis. Front. Vet. Sci. 2019; 6, 244. 10.3389/fvets.2019.00244 31404288 PMC6676220

[49] Hyde RM, Remnant JG, Bradley AJ, Breen JE, Hudson CD, Davies PL et al. Quantitative analysis of antimicrobial use on British dairy farms. The Veterinary record. 2017; 181 (25), 683. 10.1136/vr.104614 29263292

[50] van Rennings L, Münchhausen C von, Hartmann M, Ottilie H, Honscha W, Käsbohrer A et al. [Antibiotic usage and antibiotic sales in Germany in 2011—the situation of drug usage in veterinary medicine]. Berl. Münch. Tierärztl. Wochenschr. 2014; 127, 366–374. 10.2376/0005-9366-127-366 25868164

[51] Dahms C, Hübner NO, Kossow A, Mellmann A, Dittmann K, Kramer A. Occurrence of ESBL-producing *Escherichia coli* in livestock and farm workers in Mecklenburg-Western Pomerania, Germany. PloS one. 2015; 10 (11), e0143326. 10.1371/journal.pone.0143326 26606146 PMC4659621

[52] Santman-Berends IMGA Gonggrijp MA, Hage JJ Heuvelink AE, Velthuis A Lam TJGM et al. Prevalence and risk factors for extended-spectrum β-lactamase or AmpC-producing *Escherichia coli* in organic dairy herds in the Netherlands. J. Dairy Sci. 2016; 100:562–571. 10.3168/jds.2016-11839 27865491

[53] Beyer A, Baumann S, Scherz G, Stahl J, Bergen M von, Friese A et al. Effects of ceftiofur treatment on the susceptibility of commensal porcine *E*. *coli*—comparison between treated and untreated animals housed in the same stable. BMC Vet. Res. 2015; 11, 265. 10.1186/s12917-015-0578-3 26472561 PMC4608134

