# Supplementary material for: Multidrug-resistant enterobacteria in newborn dairy calves in Germany
Source: PLoS One. 2021 Mar 12;16(3):e0248291. doi: 10.1371/journal.pone.0248291 (PMC7954297; doi:10.1371/journal.pone.0248291)
Supplement: S1 Table — (DOCX) [file pone.0248291.s001.docx]

S1 Table: Primer sequences and sizes of PCR products used for the determination of *bla*-genes and the extended quadruplex phylo-typing method

| Primer Name | Sequence 5‘ 🡪 3‘ | Target | Source |
| --- | --- | --- | --- |
| CTX-M universal FWD | CGCTTTGCGATGTGCAG | all *bla*_CTX-M_ genes | Gröbner et al. (2009) |
| CTX-M universal REV | ACCGCGATATCGTTGGT |  |  |
| TEM universal FWD | ATGAGTATTCAACATTTCCG | all *bla*_TEM_ genes | Gröbner et al. (2009) |
| TEM universal REV | TTAATCAGTGAGGCACCTAT |  |  |
| SHV-MP-1 FWD | TTCGCCTGTGTATTATCTCC | *bla*_SHV-1_ genes | Gröbner et al. (2009) |
| SHV-MP-1 REV | TCCGCTCTGCTTTGTTATTC |  |  |
| CTX-M-9-MP FWD | GCAGTACAGACACAATACCG | *bla*_CTX-M-9_ genes | Gröbner et al. (2009) |
| CTX-M-9-MP REV | TATCATTGGTGGTGCCGTAG |  |  |
| CTX-M-1 FWD | CGTCACGCTGTTGTTAGGAA | *bla*_CTX-M-1_ genes | Strauß et al. (2015) |
| CTX-M-1 REV | ACGGCTTTCTGCCTTAGGTT |  |  |
| chuA.1b | ATGGTACCGGACGAACCAAC | *chuA* | Clermont et al. (2013) |
| chuA.2 | TGCCGCCAGTACCAAAGACA |  |  |
| yjaA.1b | CAAACGTGAAGTGTCAGGAG | *yjaA* | Clermont et al. (2013) |
| yjaA.2b | AATGCGTTCCTCAACCTGTG |  |  |
| TspE4C2.1b | CACTATTCGTAAGGTACTCC | TspE4.C2 | Clermont et al. (2013) |
| TspE4C2.2b | AGTTTATCGCTGCGGGTCGC |  |  |
| AceK.f | AACGCTATTCGCCAGCTTGC | *arpA* | Clermont et al. (2013) |
| ArpA1.r | TCTCCCCATACCGTACGCTA |  |  |
| ArpAgpE.f | GATTCCATCTTGTCAAAATATGCC | *arpA* (Group E-specific) | Clermont et al. (2013) |
| ArpAgpE.r | GAAAAGAAAAAGAATTCCAAGAG |  |  |
| trpAgpC.1 | AGTTTTATGCCCAGTGCGAG | *trpA* (Group C-specific) | Clermont et al. (2013) |
| trpAgpC.2 | TCTGCGCCGGTCACGCCC |  |  |
