# Supplementary material for: Multidrug-resistant enterobacteria in newborn dairy calves in Germany
Source: PLoS One. 2021 Mar 12;16(3):e0248291. doi: 10.1371/journal.pone.0248291 (PMC7954297; doi:10.1371/journal.pone.0248291)
Supplement: S2 Table — (DOCX) [file pone.0248291.s002.docx]

S2 Table: Minimum inhibitory concentrations (mg/L) determined by VITEK^®^ 2 technology for 99 ESBL-producing isolates

| **Isolate ID** | **AMP/**  **AMX** | **AMS/**  **AMC** | **TZP** | **CXM/**  **CTM** | **CPD** | **CTX/**  **CRO** | **CAZ** | **IMP** | **MEM** | **GEN** | **CIP** | **LVX** | **SXT** | **NFT** |
| --- | --- | --- | --- | --- | --- | --- | --- | --- | --- | --- | --- | --- | --- | --- |
| EC-1 | >16 | 16 | ≤4 | >32 | >4 | >32 | 4 | ≤0.25 | ≤0.25 | ≤1 | >2 | >4 | ≤20 | 64 |
| EC-2 | >16 | 16 | ≤4 | >32 | >4 | 8 | ≤1 | ≤0.25 | ≤0.25 | >8 | >2 | >4 | >160 | ≤16 |
| EC-3 | >16 | 16 | ≤4 | >32 | >4 | >32 | 4 | ≤0.25 | ≤0.25 | ≤1 | ≤0.25 | 1 | ≤20 | ≤16 |
| EC-4 | >16 | 16 | ≤4 | >32 | >4 | >32 | 4 | ≤0.25 | ≤0.25 | ≤1 | ≤0.25 | 1 | ≤20 | ≤16 |
| EC-5 | >16 | 16 | ≤4 | >65 | >4 | >32 | 4 | ≤0.25 | ≤0.25 | ≤1 | >2 | >4 | ≤20 | 64 |
| EC-6 | >16 | >16 | ≤4 | >32 | >4 | >32 | ≤1 | ≤0.25 | ≤0.25 | >8 | >2 | >4 | >160 | ≤16 |
| EC-7 | >16 | 16 | ≤4 | >32 | >4 | >32 | 4 | ≤0.25 | ≤0.25 | ≤1 | >2 | >4 | ≤20 | ≤16 |
| EC-8 | >16 | 16 | ≤4 | >32 | >32 | >4 | 2 | ≤0.25 | ≤0.25 | ≤1 | ≤0.25 | ≤0.12 | ≤20 | 32 |
| EC-9 | >16 | >16 | ≤4 | >32 | >4 | 16 | ≤1 | ≤0.25 | ≤0.25 | ≤1 | ≤0.25 | ≤0.12 | ≤20 | ≤16 |
| EC-10 | >16 | 16 | ≤4 | >32 | >4 | >32 | 4 | ≤0.25 | ≤0.25 | 8 | 0.25 | 1 | ≤20 | 16 |
| EC-11 | >16 | 16 | ≤4 | >32 | >4 | >32 | 4 | ≤0.25 | ≤0.25 | ≤1 | ≤0.25 | 1 | ≤20 | ≤16 |
| EC-12 | >16 | 16 | ≤4 | >32 | >4 | 8 | ≤1 | ≤0.25 | ≤0.25 | ≤1 | ≤0.25 | ≤0.12 | ≤20 | ≤16 |
| EC-13 | >16 | 16 | ≤4 | >32 | >4 | 8 | ≤1 | ≤0.25 | ≤0.25 | >8 | >2 | >4 | >160 | ≤16 |
| EC-14 | >16 | 16 | ≤4 | >32 | >4 | >32 | 4 | ≤0.25 | ≤0.25 | ≤1 | ≤0.25 | 1 | ≤20 | ≤16 |
| EC-15 | >16 | 16 | ≤4 | >32 | >4 | >32 | ≤1 | ≤0.25 | ≤0.25 | ≤1 | ≤0.25 | ≤0.12 | >160 | ≤16 |
| EC-16 | >16 | >16 | ≤4 | >32 | >4 | >32 | ≤1 | ≤0.25 | ≤0.25 | ≤1 | ≤0.25 | ≤0.12 | >160 | ≤16 |
| EC-17 | >16 | 16 | ≤4 | >32 | >4 | 8 | ≤1 | ≤0.25 | ≤0.25 | ≤1 | ≤0.25 | ≤0.12 | ≤20 | ≤16 |
| EC-18 | >16 | 16 | ≤4 | >32 | >4 | >32 | 4 | ≤0.25 | ≤0.25 | ≤1 | >2 | >4 | ≤20 | ≤16 |
| EC-19 | >16 | >16 | >64 | >32 | >4 | >32 | ≤1 | ≤0.25 | ≤0.25 | ≤1 | 0.5* | 1 | >160 | 64 |
| ECG-20 | >16 | 8 | ≤4 | N/A | >4 | 4 | 16 | 0.5 | ≤0.25 | ≤1 | 0.5* | 1 | ≤20 | N/A |
| EC-21 | >16 | 16 | ≤4 | >32 | >4 | >32 | ≤1 | ≤0.25 | ≤0.25 | ≤1 | ≤0.25 | ≤0.12 | ≤20 | ≤16 |
| EC-22 | >16 | 16 | ≤4 | >32 | >4 | >32 | 4 | ≤0.25 | ≤0.25 | 8 | >2 | >4 | >160 | ≤16 |
| EC-23 | >16 | >16 | ≤4 | >32 | >4 | >32 | 8 | ≤0.25 | ≤0.25 | ≤1 | >2 | 4 | ≤20 | ≤16 |
| EC-24 | >16 | >16 | ≤4 | >32 | >4 | >32 | 16 | ≤0.25 | ≤0.25 | ≤1 | >2 | 4 | ≤20 | ≤16 |
| **Isolate ID** | **AMP/**  **AMX** | **AMS/**  **AMC** | **TZP** | **CXM/**  **CTM** | **CPD** | **CTX/**  **CRO** | **CAZ** | **IMP** | **MEM** | **GEN** | **CIP** | **LVX** | **SXT** | **NFT** |
| EC-25 | >16 | >16 | ≤4 | >32 | >4 | >32 | 8 | ≤0.25 | ≤0.25 | ≤1 | >2 | 4 | ≤20 | ≤16 |
| ECG-26 | >16 | 8 | ≤4 | N/A | >4 | >32 | >32 | ≤0.25 | ≤0.25 | ≤1 | 0.5* | 1 | ≤20 | N/A |
| ECG-27 | >16 | 4 | ≤4 | N/A | >4 | 4 | 16 | 0.5 | ≤0.25 | ≤1 | 0.5* | 1 | ≤20 | N/A |
| EC-28 | >16 | 16 | ≤4 | >32 | >4 | >32 | ≤1 | ≤0.25 | ≤0.25 | ≤1 | ≤0.25 | ≤0.12 | ≤20 | ≤16 |
| ECG-29 | >16 | 4 | ≤4 | N/D | >4 | 4 | 16 | 0.5 | ≤0.25 | ≤1 | 0.5* | 1 | ≤20 | N/D |
| EC-30 | >16 | 16 | ≤4 | >32 | >4 | 8 | ≤1 | ≤0.25 | ≤0.25 | ≤1 | 0.5* | 1 | ≤20 | ≤16 |
| EC-31 | >16 | 16 | ≤4 | >32 | >4 | >32 | ≤1 | ≤0.25 | ≤0.25 | ≤1 | ≤0.25 | 1 | ≤20 | ≤16 |
| EC-32 | >16 | >16 | ≤4 | >32 | >4 | >32 | 4 | ≤0.25 | ≤0.25 | ≤1 | ≤0.25 | 0.25 | ≤20 | 64 |
| EC-34 | >16 | >16 | ≤4 | >32 | >4 | >32 | ≤1 | ≤0.25 | ≤0.25 | ≤1 | ≤0.25 | 1 | >160 | 32 |
| EC-35 | >16 | >16 | ≤4 | >32 | >4 | >32 | ≤1 | ≤0.25 | ≤0.25 | ≤1 | ≤0.25 | 1 | >160 | 32 |
| EC-36 | >16 | >16 | 32 | >32 | >4 | >32 | 16 | ≤0.25 | ≤0.25 | ≤1 | >2 | >4 | ≤20 | ≤16 |
| KP-37 | >16 | 16 | 8 | >32 | >4 | 4 | ≤1 | ≤0.25 | ≤0.25 | >8 | 1 | 1 | >160 | N/A |
| EC-38 | >16 | >16 | ≤4 | >32 | >4 | >32 | ≤1 | ≤0.25 | ≤0.25 | >8 | >2 | >4 | >160 | ≤16 |
| EC-39 | >16 | 16 | ≤4 | >32 | >4 | >32 | ≤1 | ≤0.25 | ≤0.25 | ≤1 | >2 | >4 | ≤20 | 32 |
| KP-40 | >16 | 16 | ≤4 | >32 | >4 | 4 | ≤1 | ≤0.25 | ≤0.25 | >8 | 1 | 1 | >160 | N/A |
| EC-41 | >16 | >16 | ≤4 | >32 | >4 | >32 | ≤1 | ≤0.25 | ≤0.25 | >8 | >2 | >4 | >160 | ≤16 |
| EC-42 | >16 | >16 | 16 | >32 | 4 | >32 | 16 | ≤0.25 | ≤0.25 | ≤1 | >2 | >4 | ≤20 | ≤16 |
| EC-43 | >16 | >16 | ≤4 | >32 | >4 | 8 | ≤1 | ≤0.25 | ≤0.25 | >8 | >2 | >4 | >160 | 32 |
| EC-44 | >16 | >16 | ≤4 | >32 | >4 | >32 | ≤1 | ≤0.25 | ≤0.25 | ≤1 | ≤0.25 | 1 | >160 | 64 |
| EC-45 | >16 | 16 | ≤4 | >32 | >4 | >32 | ≤1 | ≤0.25 | ≤0.25 | ≤1 | ≤0.25 | ≤0.12 | ≤20 | ≤16 |
| EC-46 | >16 | 16 | ≤4 | >32 | >4 | >32 | ≤1 | ≤0.25 | ≤0.25 | ≤1 | ≤0.25 | ≤0.12 | ≤20 | ≤16 |
| EC-47 | >16 | ≤2 | ≤4 | >32 | >4 | >32 | ≤1 | ≤0.25 | ≤0.25 | ≤1 | ≤0.25 | 1 | ≤20 | ≤16 |
| EC-48 | >16 | ≤2 | 16 | >32 | >4 | 8 | 4 | ≤0.25 | ≤0.25 | ≤1 | ≤0.25 | ≤0.12 | ≤20 | ≤16 |
| EC-49 | >16 | ≤2 | ≤4 | >32 | >4 | >32 | ≤1 | ≤0.25 | ≤0.25 | ≤1 | ≤0.25 | 1 | ≤20 | ≤16 |
| EC-50 | >16 | >16 | ≤4 | >32 | >4 | 8 | ≤1 | ≤0.25 | ≤0.25 | >8 | >2 | >4 | >160 | 32 |
| EC-51 | >16 | >16 | ≤4 | >32 | >4 | >32 | ≤1 | ≤0.25 | ≤0.25 | ≤1 | >2 | >4 | >160 | ≤16 |
| EC-52 | >16 | >16 | ≤4 | >32 | >4 | >32 | ≤1 | ≤0.25 | ≤0.25 | ≤1 | >2 | >4 | >160 | ≤16 |
| EC-53 | >16 | 16 | ≤4 | >32 | >4 | >32 | ≤1 | ≤0.25 | ≤0.25 | ≤1 | ≤0.25 | 1 | >160 | 64 |
| **Isolate ID** | **AMP/**  **AMX** | **AMS/**  **AMC** | **TZP** | **CXM/**  **CTM** | **CPD** | **CTX/**  **CRO** | **CAZ** | **IMP** | **MEM** | **GEN** | **CIP** | **LVX** | **SXT** | **NFT** |
| EC-54 | >16 | 16 | ≤4 | >32 | >4 | 8 | ≤1 | ≤0.25 | ≤0.25 | 8 | 1 | 2 | ≤20 | ≤16 |
| EC-55 | >16 | 16 | ≤4 | >32 | >4 | 32 | ≤1 | ≤0.25 | ≤0.25 | >8 | ≤0.25 | 0.5 | >160 | ≤16 |
| ECG-56 | >16 | >16 | 8 | >32 | >4 | >32 | 4 | ≤0.25 | ≤0.25 | >8 | 1 | 1 | >160 | N/A |
| EC-59 | >16 | 16 | ≤4 | >32 | >4 | >32 | 4 | ≤0.25 | ≤0.25 | ≤1 | >2 | >4 | >160 | ≤16 |
| EC-60 | >16 | 16 | ≤4 | >32 | >4 | >32 | 4 | ≤0.25 | ≤0.25 | ≤1 | >2 | >4 | >160 | ≤16 |
| EC-61 | >16 | 16 | ≤4 | >32 | >4 | 32 | 4 | ≤0.25 | ≤0.25 | ≤1 | >2 | >4 | >160 | ≤16 |
| EC-62 | >16 | ≤2 | ≤4 | >32 | >4 | >32 | 4 | ≤0.25 | ≤0.25 | ≤1 | ≤0.25 | 1 | ≤20 | ≤16 |
| EC-63 | >16 | 16 | ≤4 | >32 | >4 | >32 | ≤1 | ≤0.25 | ≤0.25 | ≤1 | ≤0.25 | ≤0.12 | ≤20 | 32 |
| EC-64 | >16 | >16 | ≤4 | >32 | >4 | >32 | 4 | ≤0.25 | ≤0.25 | >8 | >2 | >4 | >160 | 32 |
| EC-65 | >16 | 16 | ≤4 | >32 | >4 | >32 | 4 | ≤0.25 | ≤0.25 | ≤1 | >2 | >4 | >160 | ≤16 |
| EC-66 | >16 | >16 | ≤4 | >32 | >4 | >32 | 2 | ≤0.25 | ≤0.25 | ≤1 | >2 | >4 | ≤20 | ≤16 |
| EC-67 | >16 | >16 | 16 | >32 | >4 | >32 | 16 | ≤0.25 | ≤0.25 | >8 | >2 | >4 | >160 | 64 |
| EC-68 | >16 | 16 | ≤4 | >32 | >4 | 8 | ≤1 | ≤0.25 | ≤0.25 | ≤1 | ≤0.25 | 0.25 | ≤20 | ≤16 |
| EC-69 | >16 | 16 | ≤4 | >32 | >4 | >32 | ≤1 | ≤0.25 | ≤0.25 | ≤1 | ≤0.25 | ≤0.12 | ≤20 | ≤16 |
| EC-70 | >16 | 16 | ≤4 | >32 | >4 | 8 | ≤1 | ≤0.25 | ≤0.25 | ≤1 | ≤0.25 | 0.25 | ≤20 | ≤16 |
| EC-71 | >16 | 8 | ≤4 | >32 | >4 | >32 | ≤1 | ≤0.25 | ≤0.25 | >8 | >2 | 4 | >160 | 32 |
| EC-72 | >16 | 8 | ≤4 | >32 | >4 | >32 | ≤1 | ≤0.25 | ≤0.25 | >8 | >2 | 4 | >160 | ≤16 |
| EC-73 | >16 | 16 | ≤4 | >32 | >4 | >32 | ≤1 | ≤0.25 | ≤0.25 | >8 | >2 | >4 | >160 | ≤16 |
| EC-74 | >16 | >16 | 8 | >32 | >4 | >32 | 16 | ≤0.25 | ≤0.25 | >8 | >2 | >4 | >160 | ≤16 |
| EC-75 | >16 | >16 | 16 | >32 | >4 | >32 | 16 | ≤0.25 | ≤0.25 | >8 | >2 | >4 | >160 | ≤16 |
| EC-76 | >16 | >16 | 8 | >32 | >4 | >32 | 16 | ≤0.25 | ≤0.25 | >8 | >2 | >4 | >160 | ≤16 |
| EC-77 | >16 | >16 | ≤4 | >32 | >4 | >32 | ≤1 | ≤0.25 | ≤0.25 | >8 | >2 | >4 | >160 | ≤16 |
| EC-78 | >16 | >16 | ≤4 | >32 | >4 | >32 | ≤1 | ≤0.25 | 0.25 | 8 | >2 | >4 | >160 | ≤16 |
| EC-79 | >16 | 16 | ≤4 | >32 | >4 | >32 | 16 | ≤0.25 | ≤0.25 | ≤1 | >2 | >4 | >160 | ≤16 |
| EC-80 | >16 | >16 | ≤4 | >32 | >4 | >32 | 4 | ≤0.25 | ≤0.25 | ≤1 | >2 | >4 | ≤20 | ≤16 |
| EC-81 | >16 | 16 | ≤4 | >32 | >4 | >32 | ≤1 | ≤0.25 | ≤0.25 | ≤1 | ≤0.25 | 1 | >160 | 64 |
| EC-82 | >16 | 16 | ≤4 | >32 | >4 | >32 | ≤1 | ≤0.25 | ≤0.25 | ≤1 | ≤0.25 | 1 | >160 | 64 |
| EC-83 | >16 | 16 | ≤4 | >32 | >4 | 16 | ≤1 | ≤0.25 | ≤0.25 | ≤1 | ≤0.25 | 1 | ≤20 | 16 |
| **Isolate ID** | **AMP/**  **AMX** | **AMS/**  **AMC** | **TZP** | **CXM/**  **CTM** | **CPD** | **CTX/**  **CRO** | **CAZ** | **IMP** | **MEM** | **GEN** | **CIP** | **LVX** | **SXT** | **NFT** |
| EC-84 | >16 | 16 | ≤4 | >32 | >4 | 16 | ≤1 | ≤0.25 | ≤0.25 | ≤1 | ≤0.25 | 1 | ≤20 | ≤16 |
| EC-85 | >16 | 16 | ≤4 | >32 | >4 | 8 | ≤1 | ≤0.25 | ≤0.25 | ≤1 | ≤0.25 | ≤0.12 | ≤20 | ≤16 |
| EC-86 | >16 | 16 | ≤4 | >32 | >4 | 8 | ≤1 | ≤0.25 | ≤0.25 | ≤1 | ≤0.25 | ≤0.12 | ≤20 | ≤16 |
| EC-87 | >16 | 16 | ≤4 | >32 | >4 | >32 | 16 | ≤0.25 | ≤0.25 | ≤1 | ≤0.25 | ≤0.12 | >160 | 64 |
| EC-88 | >16 | ≤2 | ≤4 | >32 | >4 | >32 | 4 | ≤0.25 | ≤0.25 | ≤1 | >2 | >4 | ≤20 | 128 |
| EC-89 | >16 | 16 | ≤4 | >32 | >4 | 16 | ≤1 | ≤0.25 | ≤0.25 | ≤1 | ≤0.25 | 1 | ≤20 | 32 |
| EC-90 | >16 | >16 | ≤4 | >32 | >4 | >32 | 8 | ≤0.25 | ≤0.25 | ≤1 | >2 | 4 | >160 | ≤16 |
| EC-91 | >16 | >16 | >64 | >32 | >4 | >32 | 2 | ≤0.25 | ≤0.25 | ≤1 | 0.5* | 1 | >160 | 64 |
| EC-92 | >16 | >16 | ≤4 | >32 | >4 | >32 | 4 | ≤0.25 | ≤0.25 | ≤1 | 0.5* | 1 | ≤20 | ≤16 |
| EC-93 | >16 | 16 | ≤4 | >32 | >4 | 32 | 4 | ≤0.25 | ≤0.25 | 8 | >2 | >4 | >160 | ≤16 |
| EC-94 | >16 | 16 | ≤4 | >32 | >4 | >32 | ≤1 | ≤0.25 | ≤0.25 | ≤1 | ≤0.25 | ≤0.12 | ≤20 | ≤16 |
| EC-95 | >16 | 16 | ≤4 | >32 | >4 | >32 | 2 | ≤0.25 | ≤0.25 | 4 | >2 | >4 | >160 | ≤16 |
| EC-96 | >16 | 16 | ≤4 | >32 | >4 | >32 | ≤1 | ≤0.25 | ≤0.25 | ≤1 | ≤0.25 | ≤0.12 | ≤20 | ≤16 |
| EC-97 | >16 | 16 | ≤4 | >32 | >4 | >32 | 4 | ≤0.25 | ≤0.25 | ≤1 | ≤0.25 | 1 | ≤20 | ≤16 |
| EC-98 | >16 | 16 | ≤4 | >32 | >4 | 8 | ≤1 | ≤0.25 | ≤0.25 | ≤1 | ≤0.25 | ≤0.12 | ≤20 | ≤16 |
| EC-99 | >16 | 16 | ≤4 | >32 | >4 | 8 | ≤1 | ≤0.25 | ≤0.25 | ≤1 | ≤0.25 | ≤0.12 | ≤20 | ≤16 |
| EC-100 | >16 | >16 | ≤4 | >32 | >4 | 8 | ≤1 | ≤0.25 | ≤0.25 | ≤1 | ≤0.25 | ≤0.12 | ≤20 | ≤16 |
| EC-101 | >16 | 16 | ≤4 | >32 | >4 | 16 | ≤1 | ≤0.25 | ≤0.25 | ≤1 | ≤0.25 | 0.5 | >160 | ≤16 |
| EC-102 | >16 | >16 | ≤4 | >32 | >4 | >32 | ≤1 | ≤0.25 | ≤0.25 | ≤1 | >2 | >4 | >160 | ≤16 |

Values which were interpreted as susceptible are shaded in light green; those interpreted as resistant are shaded in light red; MICs that are not coloured, indicate intermediary susceptibility; * MIC in area of technical uncertainty

EC – *E. coli*, ECG – *Enterobacter cloacae* group, KP – *Klebsiella pneumoniae* subsp. *pneumoniae*; AMP – ampicillin, AMX – amoxicillin, AMS – ampicillin-sulbactam, AMC – amoxicillin-clavulanic acid, TZP – piperacillin-tazobactam, CXM – cefuroxime, CTM – cefotiam, CPD – cefpodoxime, CTX – cefotaxime, CRO – ceftriaxone, CAZ – ceftazidime, IMP – imipenem, MEM – meropenem, GEN – gentamicin, CIP – ciprofloxacin, LVX – levofloxacin, SXT – trimethoprim-sulfamethoxazole, NIT – nitrofurantoin
