# Supplementary material for: Multidrug-resistant enterobacteria in newborn dairy calves in Germany
Source: PLoS One. 2021 Mar 12;16(3):e0248291. doi: 10.1371/journal.pone.0248291 (PMC7954297; doi:10.1371/journal.pone.0248291)
Supplement: S3 Table — (DOCX) [file pone.0248291.s003.docx]

S3 Table: Gross amounts of antibiotic substance classes (in g) used on the farms in total and in newborn calves

|  | Farm 1 | | Farm 2 | | Farm 3 | | Farm 4 | | Farm 5 | | Farm 6 | | Farm 7 | | Farm 9 | | Farm 10 | |
| --- | --- | --- | --- | --- | --- | --- | --- | --- | --- | --- | --- | --- | --- | --- | --- | --- | --- | --- |
|  | Total | Calves | Total | Calves | Total | Calves | Total | Calves | Total | Calves | Total | Calves | Total | Calves | Total | Calves | Total | Calves |
| **AMG** | 237.7 | 0.0 | 161.3 | 2.8 | 380.8 | 135.5 | 220.1 | 0.0 | 260.2 | 0.0 | 74.3 | 0.0 | 900.3 | 381.3 | 315.5 | 19.3 | 85.5 | 0.0 |
| **PEN** | 8241.1 | 4.2 | 4356.0 | 23.4 | 15575.8 | 429.5 | 14571.9 | 738.7 | 13652.4 | 125.6 | 2486.7 | 25.8 | 21744.4 | 1409.5 | 6785.6 | 486.6 | 3004.7 | 227.3 |
| **CEP** | 1557.9 | 0.0 | 210.2 | 0.2 | 437.8 | 0.5 | 454.9 | 0.0 | 338.7 | 0.0 | 102.1 | 0.0 | 1617.7 | 0.0 | 3011.4 | 0.0 | 22.1 | 0.0 |
| **AMP** | 93.6 | 0.0 | 175.2 | 36.6 | 256.5 | 135.0 | 3304.8 | 526.2 | 25.5 | 18.0 | 98.8 | 42.8 | 3280.8 | 74.1 | 379.1 | 40.7 | 412.5 | 36.9 |
| **FLQ** | 521.9 | 0.0 | 437.7 | 28.4 | 1268.4 | 141.6 | 1097.0 | 8.0 | 797.0 | 0.0 | 10.4 | 0.0 | 1014.7 | 137.0 | 1498.1 | 19.5 | 696.6 | 27.0 |
| **TRI** | 89.4 | 0.0 | 186.5 | 1.6 | 455.7 | 12.7 | 165.6 | 0.0 | 0.0 | 0.0 | 21.4 | 1.8 | 809.9 | 182.9 | 83.0 | 0.0 | 256.8 | 0.0 |
| **ION** | 1620.0 | 0.0 | 0.0 | 0.0 | 0.0 | 0.0 | 0.0 | 0.0 | 0.0 | 0.0 | 0.0 | 0.0 | 97.2 | 0.0 | 0.0 | 0.0 | 0.0 | 0.0 |
| **LIN** | 0.0 | 0.0 | 0.0 | 0.0 | 7.9 | 0.0 | 108.2 | 0.0 | 157.1 | 0.0 | 0.0 | 0.0 | 125.2 | 72.8 | 51.2 | 0.0 | 0.0 | 0.0 |
| **MAC** | 2.6 | 0.3 | 6.9 | 1.7 | 100.8 | 0.0 | 66.9 | 1.3 | 1654.0 | 25.1 | 8.3 | 2.9 | 5597.9 | 0.0 | 42.0 | 0.2 | 0.0 | 0.0 |
| **PLM** | 0.0 | 0.0 | 0.0 | 0.0 | 0.0 | 0.0 | 0.0 | 0.0 | 0.0 | 0.0 | 0.0 | 0.0 | 0.0 | 0.0 | 0.0 | 0.0 | 0.0 | 0.0 |
| **PLP** | 0.0 | 0.0 | 15.8 | 7.7 | 224.5 | 211.3 | 0.0 | 0.0 | 0.0 | 0.0 | 0.0 | 0.0 | 0.0 | 0.0 | 0.0 | 0.0 | 0.0 | 0.0 |
| **SUL** | 446.8 | 0.0 | 932.6 | 8.2 | 2278.4 | 63.6 | 828.0 | 0.0 | 0.0 | 0.0 | 107.0 | 9.0 | 4049.5 | 914.3 | 2178.8 | 0.0 | 1284.0 | 0.0 |
| **TET** | 2495.3 | 0.0 | 1585.1 | 0.0 | 4482.7 | 110.9 | 1320.0 | 0.0 | 1259.7 | 0.0 | 167.2 | 0.0 | 9933.4 | 1.3 | 640.5 | 0.0 | 104.9 | 2.5 |

AMG – aminoglycosides, PEN – penicillins, CEP – cephalosporins, AMP – amphenicols, FLQ – fluoroquinolones, TRI – trimethoprim, ION – ionophores, LIN – lincosamides, MAC – marolides, PLM – pleuromutilins, PLP – polypeptides, SUL – sulfonamides, TET – tetracyclines
